# The Prevalence of Exposure to Domestic Violence Among High School Students in Tehran

**DOI:** 10.5812/ircmj.13246

**Published:** 2014-01-05

**Authors:** Homeira Sajadi, Hossein Rahimy, Hassan Rafiey, Meroe Vameghi

**Affiliations:** 1 National Board on Social Medicine, Social Determinants of Health Research Center, University of Social Welfare and Rehabilitation Sciences, Tehran, IR Iran; 2 Department of Social Work, University of Social Welfare and Rehabilitation Sciences, Tehran, IR Iran; 3 Social Welfare Educational Group, University of Social Welfare and Rehabilitation Sciences, Tehran, IR Iran; 4 Social Welfare Management Research Center, University of Social Welfare and Rehabilitation Sciences, Tehran, IR Iran

**Keywords:** Domestic Violence, Prevalence, Students

## Abstract

**Background:**

Domestic violence appears to be a major social problem. Researches in the last 10 years have uncovered multiple effects of witnessing domestic violence on children, ranging in severity from little or no effect to sever psychological harm.

**Objectives:**

This study aimed to measure the prevalence of exposure to domestic violence among high school students in Tehran.

**Materials and Methods:**

A cross-sectional survey was conducted on high school students of Tehran in the school year 2011–2012. The “Children’s Exposure to Domestic Violence Scale” was administered to a total cohort of 1,212 students (615 males and 597 females) selected by the stratified sampling method.

**Results:**

Approximately one-half of the participants (44.3%) had been exposed to their fathers’s violence against their mothers at least sometimes in their lives, the most common form of which was preventing the mother from doing something (28.5%) and the least common, hurting the mother with sharp or deadly tools (9.6%). A substantial proportion of the students (90.6%) had been exposed to violence in the community or at school, the most common kind would be being heard from someone calling another person names or making fun of them (81.7%) and the least common, being injured a child in the community or at school (31.8%).

**Conclusions:**

Exposure to violence is a widespread problem among children in Tehran. It encompasses a wide range and children were exposed to violence in different ways and forms.

## 1. Background

Among the types of violence against women, the most common has been domestic violence perpetrated by the women’s husband or partner (1), and it has been defined as “Any incident of threatening behavior, violence, or abuse (psychological, physical, sexual, financial, or emotional) between adults who are or have been intimate partners or family members regardless of gender or sexuality” (2). Violence between couples has now become part of family life and seems to be growing day by day (3). Some recent estimates indicated that at least one-fourth of women have experienced one form of physical violence in their lifetime (4). In the U.S., approximately three million families experienced extreme violence at least once during a year. Taking into account the average of two children in every home, at least 3.3 million children were exposed to violence between parents (3). According to UNICEF, annually 133–275 million children worldwide witness domestic violence (5).

Researchers, policy makers, and practitioners have used several different terms to define children’s exposure to adult domestic violence (6). The terms “witnesses” and “observers” of violence have been frequently used (6), but these terms are replaced by an expanded terminology referring to child “exposure” to domestic violence. Exposure can include watching or hearing about violent events, direct involvement (for example, trying to intervene or calling the police), or experiencing the aftermath (for example, observing bruises or observing maternal depression) (7). Edleson (3) considered the concept of children’s exposure to domestic violence beyond the home environment, and in a comprehensive scale introduced any kind of violence in the surrounding environment, including school, community, movies, and video games, within a general implication of domestic violence.

Domestic violence in Iran appears to be a major social problem (8). Based on the national survey entitled “Investigating Domestic Violence against Women in 28 Provinces of the Country” (9), primary physical violence (since the beginning of marital life) and secondary physical violence (from mid-life onwards) on a national scale were estimated as 81.7 and 62.2 percent, respectively. The same study reported that severe physical violence had a prevalence of 30%. Bearing these figures in mind, it is incontrovertible that considerable population of Iranian children is exposed to violence between their parents. As Vameghi et al. (8) asserted, 22.8 percent of the high school students in Tehran were exposed to physical violence between their parents.

Researches in the last 10 years (10) have uncovered multiple effects of witnessing domestic violence on children, ranging in severity from little or no effect to severe psychological harm. Edleson (3) reviewed over 80 studies related to violence and grouped the problems of children exposed to domestic violence into three main categories: (a) behavioral and emotional functioning problems; (b) cognitive functioning and attitude problems; and (c) physical functioning problems. Furthermore, Margolin and Gordis’ review (11) showed that children’s exposure to domestic violence may lead to a wide range of short-term and long-term consequences. The short-term consequences include aggression and delinquency; emotional and mood disorders; post-traumatic stress syndrome, such as exaggerated startle and nightmares; physical symptoms, such as sleep disturbance; and cognitive and academic problems. On the other hand, violence in the long-term may predispose children to become a victim or perpetrator of violence later in life. It also seems that children exposed to parental violence experience pernicious effects on their relationships with their parents, personality development, coping style, tendency towards violence, academic success, social well-being, and compatibility, which are to be dealt with separately in another paper in progress.

Despite its importance, few studies in Iran have been conducted on the prevalence of children’s exposure to parental violence. Vameghi et al. (8) investigated the prevalence of children’s exposure to physical aggression by their parents, and Shahedifar et al. (12) showed the relation between exposure to physical aggression by parents and self-esteem in university students. While these two studies only took account of physical aggression by parents using a researcher-designed questionnaire, the instrument used in this study is a recently defined, comprehensive, and standard questionnaire, based on a new definition of exposure capable of assessing exposure to physical violence, in addition to exposure to other aspects of domestic violence, including parents arguing, psychological abuse, violence in the neighborhood, school, and media, violence directly experienced by children, and risk factors.

## 2. Objectives

This study aimed to describe the prevalence of exposure to domestic violence in Iranian children by demographic characteristics, and to compare males’ with females’ frequency of exposure to violence using subscales of the questionnaire.

## 3. Materials and Methods

This study is a cross-sectional survey, conducted on 19 educational districts of Tehran, the capital of Iran, in 2012. The sample, taking into consideration 0.05 acceptable errors for type I errors, was estimated to consist of 1200 persons.

As Edleson, the designer of scale, administered it to populations below the age of 16, we selected an initial cohort of 1320 high school students from the first grade and second grade, of whom 108 declined to participate and 1212 consented. The stratified sampling was conducted by assuming each educational district and gender as the strata. The weight of each stratum in the sample was determined in proportion to the total sample. After that, the gender fraction of each district’s students was multiplied by the number of members from that district, hence the number of each district’s sample in terms of gender. Next, with regard to the calculated sample population of each district for each gender, one or two schools were randomly selected from a list of the districts’ schools and, from each school, one or more first grade and second grade classes (until satisfying the sample required) were randomly selected.

The “Child Exposure to Domestic Violence Scale (CEDV)” (13) was utilized in this study. In the original version, the CEDV subscales showed relatively high Cronbach’s alphas ranging from α = 0.50 to 0.76; overall α was 0.84. The test–retest reliability for each subscale was found ranging from 0.57 to 0.70, all of them statistically significant at P < 0.001. The scale’s convergent validity scores, compared with TISH (Things I have Heard and Seen), which is designed to measure the same construct, were tallied to be statistically significant, and a positive correlation existed both at the level of home (r = 0.494, P < 0.001) as well as community violence exposure (r = 0.397, P < 0.001). The CEDV scale has been rendered into several languages and administered. Additionally, in the present study, the English version was first translated into Persian by two Persian translators and then translated back into English by two other translators. After the final confirmation by the original designer, the reliability and validity of the Persian version were examined in the Iranian population (14). Exploratory factor analysis of the Persian version revealed that the scale measured seven subscales. In order to assess the concurrent validity of the scale, the Pearson correlation coefficients between the scores of the CEDV and of the questionnaire on exposure to physical aggression (8) were calculated. The results displayed a significant and positive correlation between the two subscales of exposure to parental violence (r = 0.54, P < 0.001), and the starting time of exposure to parental violence (r = 0.89, P < 0.001) between the two measures. To assess the reliability of the scale, the test–retest method was used. The Pearson correlation coefficients between the two consecutive measurements for the subscales were 0.58 to 0.89, and for the total scale it was 0.86 (P < 0.001). In addition, none of the subscales or the scale showed a significant difference between the two measurement times (p > 0.05). The internal consistency of the subscales and the scale was measured using Cronbach’s alpha coefficient, which was 0.69 to 0.83 for the subscales and 0.89 for the total scale.

The questionnaire was self-administered. Before embarking on the research, the questionnaire was approved by the Ethics Committee of the University of Social Welfare and Rehabilitation Sciences dated March 4, 2013 (91/801/T/29769). Students’ consent to participate in the research was obtained verbally; prior to handing out the questionnaires, the students were told clearly that “some of the questions ask you very private matters; therefore, you have no obligation to participate in the study, and at any time you can exit the research.” Then, an explanation about the project and on how to complete the questionnaire was given verbally to each participant, who, if willing, received a questionnaire. It took 30 minutes at most to complete. Before every question, except those related to the manner of exposure to domestic violence, there was a set of four frequency marks, namely never = 0, sometimes = 1, often = 2, and almost always = 3. To determine the exposure or non-exposure of the participants, the two options given were never = 0 (no exposure) and at least sometimes = 1 (exposure); this means that if any participant, at least once in his/her lifetime, had been exposed to one of the forms of violence between parents, he/she was considered as “exposed” and otherwise as “not exposed”. There was no necessity to provide names or identification signs. To prevent any sensitivity, all the students in a class were asked to participate, unless they were unwilling. The data were analyzed using SPSS statistical software to obtain descriptive and analytical statistics and calculate the frequency, mean, standard deviation, and chi-square.

## 4. Results

The sample comprised 1,212 first-grade and second-grade high school students in Tehran, of whom 615 (50.7%) were male and 597 (49.3%) were female (Table 1). 

**Table 1.** Distribution of Participants by District and Gender

District	Gender		Total, No. (%)
	Female	Male	
**1**	40	36	76 (6.3)
**2**	46	48	94 (7.8)
**3**	27	28	55 (4.5)
**4**	59	61	120 (9.9)
**5**	55	58	113 (9.3)
**6**	26	30	56 (4.6)
**7**	18	20	38 (3.1)
**8**	34	35	69 (5.7)
**9**	23	22	45 (3.7)
**10**	19	20	39 (3.2)
**11**	19	23	42 (3.5)
**12**	31	34	65 (5.4)
**13**	19	18	37 (3.1)
**14**	36	39	75 (6.2)
**15**	44	36	80 (6.6)
**16**	24	26	50 (4.1)
**17**	21	17	38 (3.1)
**18**	34	44	78 (6.4)
**19**	22	20	42 (3.5)
**Total**	597	615	1212 (100)

The average age of boys, girls, and all the participants were 15.52 years (SD = 0.78), 15.58 years (SD = 0.65), and 15.55 years (SD = 0.72), respectively. The mean of the grade point average (min. = 0, max. = 20) of the students was 17.67 (SD = 1.86) and the average area of their homes was 97.63 square meters (SD = 47.04). Almost all the participants (98.2%) were living in their own homes (either private or rental). A total of 1,139 of them (94.0%) stated that they lived with their fathers, 1,191 (98.3%) with their mothers, and 895 (73.8%) with their other siblings. Only a minority of 15 participants (1.2%) had a stepfather or stepmother and just 50 participants (4.1%) stated living also with their grandfathers or grandmothers. The education level of the parents ranged from illiteracy to a doctorate, but the majority (34.5% of the fathers and 47.4% of the mothers) had a high school diploma. Only 14 fathers (1.2%) and 21 mothers (1.7%) were illiterate. Moreover, 15 fathers (1.2%) and 5 mothers (0.4%) held a doctoral degree. Nearly all the participants (92.2%) stated that their parents were living together, 58 parents (4.8%) declared the divorce, and 36 respondents (0.3%) the death of one of their parents (Table 2). As for their parents’ conflicts, 553 (45.6%) claimed that they did not remember the parents’ fighting and 287 (23.7%) mentioned that they had been fighting for as long as they could remember.

**Table 2.** Demographic Characteristics of the Participants

Variable	Mean ± SD	No. (%)
**Age of children**	15.55 ± 0.72	1212 (100)
**Grade point average**	17.67 ± 1.86	1167 (96.3)
**Home’s area**	97.63 ± 47.04	1096 (90.4)
**Where the child lived**		
Our house (private or rented)	-	1190 (98.2)
Home of relatives or acquaintances	-	21 (1.7)
Shelter	-	1 (0.1)
**People the child lived with (multiple answers possible)**		
Father	-	1139 (94)
Mother	-	1191 (98.3)
Grandparent	-	50 (4.1)
Step-parent	-	15 (1.2)
Sibling	-	895 (73.8)
**Fathers’ education**		
Illiterate	-	14 (1.2)
Basic reading and writing	-	16 (1.3)
Less than high school diploma	-	355 (29.3)
High school diploma	-	418 (34.5)
Association diploma (a 2-year degree course)	-	102 (8.4)
BS/BA	-	185 (15.3)
MS/MA	-	50 (4.1)
Doctorate	-	15 (1.2)
Missing	-	57 (4.7)
**Mothers’ education**		
Illiterate	-	21 (1.7)
Basic reading and writing	-	19 (1.6)
Less than high school diploma	-	325 (26.8)
High school diploma	-	574 (47.4)
Association diploma (a 2-year degree course)	-	99 (8.2)
BS/BA	-	118 (9.7)
MS/MA	-	19 (1.6)
Doctorate	-	5 (0.4)
Missing	-	32 (2.6)
**Parents’ communication situation**		
Parents living together	-	1118 (92.2)
Parents have divorced	-	58 (4.8)
One parent has died	-	36 (0.3)
**Starting time of conflict between parents**		
I don’t remember them fighting	-	553 (45.6)
From this year	-	53 (4.4)
From 2–3 years ago	-	178 (14.7)
At least 4 years ago or earlier	-	132 (10.9)
For as long as I can remember	-	287 (23.7)
Missing	-	9(0.7)

Table 3 presents information on the subscales of the questionnaire. Exposure to parental conflicts, as one form of domestic violence, had a very high prevalence: 1010 children (83.3%) had at least sometimes been exposed to in their lifetime (“had been exposed at least sometimes in their lifetime” is referred to as “exposed” after this point). The most common and rarest forms were adults’ disagreement (69.3%) and father’s emotional abuse of mother (35.6%), respectively.

Nearly one-half of the children (44.3%) were exposed to one form of the father’s violence against the mother, the most common of which was when the father prevented the mother from doing something (n = 346, 28.5%) and the least common form, on the other hand, was the father’s injuring the mother with sharp or deadly objects, such as a knife (n = 116, 9.6%). 

More than one-half of the children (57.9%) became involved -in some way- in the father’s violence against mother or inter-parental violence. The most and least common forms were yelling at their parents during the fighting in a different room (n = 542, 44.7%), and the father’s abusing the child to hurt the mother (13.4%), respectively.

With regard to the family risk factors, 680 (56.1%) were exposed to some forms. The most common risk factor was the occurrence of major life changes, such as moving home, hospitalization, divorce, death of a family member, or incarceration of a parent (n = 616, 50.8%), whereas the least common form was the mother lapsing into unconsciousness due to substance abuse (15.2%).

Exposure to violence in the community or at school was also highly prevalent: 1,098 participants (90.6%) stated they had been exposed to some form of violence, the most common form of which was when the child heard someone calling others names or making fun of them (n = 990, 81.7%). The least common form of violence in this regard was when someone hurt the child in one way or another at school or in the community (n = 385, 31.8%). Not surprisingly, the highest prevalence of exposure to violence was witnessing someone being injured or killed in films or video games (n = 1195, 98.6). Just above one-half of the children studied (54.5%) revealed that they had experienced one form of adult violence against them. The most common form of adult violence against the children was hurting the child emotionally (n = 552, 45.5%), while the least common form was sexual abuse, with 112 patients (9.2%) claiming exposure at least sometimes in their lifetime.

**Table 3.** Relative Frequency of the Subscales

Variable	Never	Sometimes	Often	Almost Always
**Exposure to parental conflicts**	43.06	41.7	10.8	4.44
**Elders disagree with each other in your family**	30.7	52.7	11.8	4.8
**Your father hurt your mother’s feeling**	64.4	26.7	5.9	2.9
**Your father and mother argue with each other over you**	34.1	45.6	14.7	5.6
**Exposure to the father’s violence against the mother**	81.28	16.41	1.7	0.3
**Your father stopped your mother from doing something**	71.5	24.4	2.3	1.8
**Your father stopped your mother from eating or sleeping**	82.8	15.9	0.7	0.6
**Your father broke or destroyed something on purpose**	76.2	20.1	3.2	0.4
**Your father hurt your mother’s body**	80.0	16.7	2.7	0.5
**Your father threatened your mother that he would use sharp or killing tools to hurt her**	86.8	12.2	0.8	0.2
**Your father really hurt your mother by using sharp or killing tools**	90.4	9.2	0.3	0.0
**Child’s involvement in parents’ violence**	73.0	21.6	4	1.4
**Your parents started fighting in “another room” and you yelled at them**	55.3	36.6	6.3	1.9
**Your parents started fighting and you asked somebody else to help you**	73.3	21.2	3.8	1.7
**You physically engaged in your parents’ fighting**	70.6	21.6	5.1	2.6
**Your father did something to you to hurt or threaten your mother**	86.6	12.0	1.2	0.2
**You tried to escape from where your parents’ fighting was taking place**	78.5	16.5	4.0	1.0
**Your father asked you to tell him what your mother had told you**	73.3	21.6	4.0	1.0
**Family risk factors**	70.45	22.0	4.1	3.45
**You worry that your father is disturbed by using a substance**	77.4	15.8	2.6	4.3
**You worry that your mother is disturbed by using a substance**	84.8	10.9	1.7	2.6
**You think big changes have happened throughout your life**	49.2	39.4	8.0	3.5
**Exposure to violence at school or in the community**	46.7	36.6	11.0	5.7
**You heard someone in your neighborhood or your school annoy other people by making fun of them or calling them names**	18.3	41.6	23.4	16.7
**Someone in your neighborhood or your school annoyed you by making fun of you or calling you names**	48.3	39.6	9.7	2.3
**You annoyed someone purposefully by calling them names or making fun of them**	52.0	35.1	7	5.9
**You hurt someone physically, by hitting, kicking, or similar activities**	62.5	26.7	7.8	3.1
**You have seen someone get hurt in your neighborhood or your school**	30.8	48.9	14.6	5.7
**You have been hurt by others in your neighborhood or your school**	68.2	27.7	3.7	0.3
**Exposure to violence through the use of violent video technologies**	5.05	21.9	31.95	41.1
**You saw in TV or movies that someone got hurt or was killed**	1.6	16.1	36.1	46.2
**You saw in a visual game (like PlayStation) that someone got hurt or was killed**	8.5	27.7	27.8	36.0
**Adult violence against the child**	74.55	18.2	4.75	2.5
**An adult in your family hurt your feelings**	54.5	26.0	12.9	6.7
**An adult in your family did something to hurt your body**	74.4	20.3	2.6	2.6
**Someone, “not your family members,” had an immoral relationship with you**	78.5	18.6	2.4	0.6
**Someone, “among your family members,” had an immoral relationship with you**	90.8	8.0	1.1	0.2

Table 4 shows the frequency of various forms of children’s exposure to father’s violence against the mother. The manner of exposure differs depending on the type of violence perpetrated. When the father prevented the mother from engaging in her favorite activity, eating, or sleeping; broke something advertently; or hurt the mother physically, the child was often an eyewitness. In other cases, the child heard about violence already committed, such as when the father threatened the mother, used sharp or deadly objects with intent to injure her, or hurt her physically with an object. 

Overall, 35.33 percent of the children had directly observed the violence occurred, while 7.02 percent witnessed violence from a different room.

**Table 4.** Frequency of Forms of Children’s Exposure to Father’s Violence against Mother ^a^

Variable	Saw the outcome	Heard about it afterwards	Heard it while it was happening	Saw it from far away	Saw it from nearby
**Forms of Children’s Exposure to the Father’s Violence against the Mother ^b^**					
**Your father stopped your mother from doing something**	19 (7.5)	127 (36.7)	75 (21.7)	7 (2.0)	111 (32.1)
**Your father stopped your mother from eating or sleeping**	16 (7.7)	63 (30.3)	44 (21.2)	23 (11.1)	62 (29.8)
**Your father broke or destroyed something on purpose**	52 (18.1)	27 (9.4)	69 (24.0)	8 (2.8)	132 (45.8)
**Your father hurt your mother’s body**	39 (16.1)	34 (14.0)	28 (11.6)	29 (12)	112 (46.3)
**Your father threatened your mother that he would use sharp or killing tools to hurt her**	14 (8.8)	57 (35.6)	36 (22.5)	9 (5.6)	44 (27.5)
**Your father really hurt your mother by using sharp or killing tools**	43 (37.1)	32 (27.6)	5 (4.3)	19 (16.4)	17 (14.7)
**Total**	183 (13.52)	340 (25.13)	257 (19.0)	95 (7.02)	478 (35.33)

^a^ data are expressed as No. (%)

^b^ Every child for each question had only to choose one option

Table 5 compares males’ with females’ frequency of exposure to violence according to seven subscales. The female participants were significantly more exposed to parental violence compared to male participants (P = 0.018, x^2^ = 5.61). In addition, the female children were significantly more involved in inter-parental violence (P = 0.003, x^2^ = 8.61). However, there was no significant difference between males and females in terms of exposure to parental conflicts, violence in the community or at school, and family risk factors. Significantly more boys than girls used and were exposed to violent films or video games (P = 0.024, x^2^ = 5.1), whereas significantly more girls than boys had experienced adult violence against themselves (x^2^ = 1.21, P < 0.001).

**Table 5.** Comparison of Children’s Exposure to Subscales of Violence by Gender

Variable	Boy		Girl		X^2^	P Value
	Never, No. (%)	At least sometimes, No. (%)	Never, No. (%)	At least sometimes, No. (%)				
**Exposure to parental conflicts**	45 (7.3)	570 (92.7)	42 (7)	555 (93)	0.03	0.849
**Exposure to the father’s violence against the mother**	363 (59)	252 (41)	312 (52.3)	285 (47.7)	5.61	0.018
**Child’s involvement in parents’ violence**	284 (46.2)	331 (53.8)	226 (37.9)	371 (62.1)	8.61	0.003
**Family risk factors**	267 (43.4)	384 (56.6)	265 (44.4)	332 (55.6)	0.11	0.733
**Exposure to violence at school or in the community**	52 (8.5)	563 (91.5)	62 (10.4)	535 (89.6)	1.32	0.25
**Exposure to violence through the use of violent video technologies**	4 (0.7)	611 (99.3)	13 (2.2)	584 (97.8)	5.1	0.024
**Adult violence against the child**	375 (61)	240 (39)	176 (29.5)	421 (70.5)	12.1	< 0.001

## 5. Discussion

The prevalence of violence by father against the mother, at least sometimes in their life, was about 41 percent for the boys, 47.7 percent for the girls, and 44.3 percent for the whole sample. The results of this study show greater prevalence of children’ exposure to domestic violence than that of the previous studies conducted in Iran. For example, Shahedifar et al. (12) reported the figures 25.8, 45.5, and 35.6 percent for the male, female, and total exposure of university students to parental violence, respectively. Vameghi et al.’s (8) study revealed 31.2 percent for girls, 16.1 percent for boys, and 22.8 percent for the total students. This discrepancy might be be due to application of different definitions of the term “domestic violence” as well as different questionnaires. While both of the previous studies measured only physical dimension of violence between parents, the present study encompassed mental violence, too, such as preventing the mother from eating, sleeping, or carrying out her favorite activity, or displaced aggression, such as breaking objects on purpose.

Significantly more girls than boys were exposed to the father’s violence against the mother. Vameqi et al. (8) attributed the finding that girls had been exposed to violence approximately twice as much as boys to the greater presence of girls at home. In addition, this mighte be accounted for by culture; the presence of male children at home may have deterrent effects on the father’s violence. In fact, the masculine characteristics of boys may spur them into action upon incidents of violence against their mothers. The presence of girls, on the other hand, may not.

The most common manners of exposure to father’s violence against the mother in this study were observing the incidents (35.33%) and hearing about the violence (25.13%). Previous researchs have yielded similar results. For example, Vamaqi et al. (8) reported that the participants were exposed to parental physical violence by witnessing (59.8%) and hearing about (21.5%). In addition, depending on the type of violence, the manners of exposure varied as well. Such minor cases of violence as preventing the mother from doing something were mostly heard about afterwards rather than witnessed, simultaneously. This might be due to the fact that these types of violence are mental rather than physical, and that they mostly occur in the parents’ privacy. In more severe cases, such as when the father was breaking objects or hurting the mother physically, the main type of exposure was witnessing the incident, personally. Finally, when the most extreme types of violence (such as injuring the mother with objects) occurred, it seems that the children were not present, and recognized the violence later by observing bruises or cuts.

Nearly one-half of the participants (57.9%) were involved -in some way- in the violence of the father against the mother or inter-parental conflicts, with girls being significantly more involved than boys. The most common type was intervening by yelling from a different room. Because the girls’ exposure to parental violence was greater compared to the boys’, it is reasonable to expect more interference. Another explanation might be proposed as physical incapability of girls to take any other action than crying or shouting.

Concerning the risk factors leading to domestic violence, 56.1 percent declared exposure at least sometimes in their lifetime. The most common form was worrying about major changes, such as moving home. Since a considerable number of Tehranians live in rental homes and have to move once a year or so, it is quite likely that most children in Tehran undergo this change. In the long run, this issue can entangle all the members of a family for a certain period, both before and after settling down. These vicissitudes can bring about great anxiety about termination of the contract, searching for a new place, or even ending up homeless.

Another risk factor, though of slight significance, was alcohol abuse. Only a tiny percentage of the participants admitted to alcohol abuse in their families. In the same vein, another study estimated that only 0.5 to 0.75 percent of women in Iran abused drugs among them the prevalence of alcohol abuse was even lower (0.2%) (15). This might be due to the pertinent Islamic teachings and conventional taboos and, on the other hand, severe criminal penalties. Therefore, it can safely be deduced that, at least for the study population, the drug and alcohol abuse were not important risk factors.

Exposure to violence in the community or at school was reported by 90.6 percent of the whole sample studied. The majority of the respondents claimed that they had seen the incidents happening, whereas a small minority stated that they had been subjected to violence personally or they had been the perpetrator themselves. Likewise, Edleson et al. (6) reported that 41 percent of his respondents had at least sometimes and 51 percent had often been exposed to hearing someone calling another names. The findings of this research are consistent with those of previous studies in the field. Richters and Martinez (16) showed that 72 percent of the children in Washington had observed a form of violence, and Osofsky et al. (17) reported that approximately 91 percent of the fifth graders in a New Orleans school had witnessed a form of violence. Bell and Jenkins (18) studied an African-American sample of schoolchildren in Chicago and found that one-third had witnessed at least one suicide and two-thirds an aggressive assault. There was no significant difference between male and female samples.

Playing violent video games and watching films had the highest prevalence among the children studied. Practically, all of them (98.6%) stated that they had been exposed to this form of violence, which is no surprise in the modern world. Video games have enthralled children, especially in the recent decade, though at the expense of exposure to violent behavior or scenes and wasted time. These adverse effects are comparable to those of watching TV films. In general, it was suggested that violence, pornography, excitement, and comedy were four factors attracting an audience. In Iran, however, the depiction of nudity and sex is prohibited by law. As an obvious result, relatively more violence can be expected on Iranian TV, hence the increased chance of children’s exposure. 

More than one-half of the children participating in this study reported adult violence against themselves. The types of violence varied, ranging from emotional hurt (45.5%) to sexual abuse by a family member (9.2%). The prior research that investigated child abuse lends support to the current study. Khushabi et al. (19) found that there was a prevalence of 62.5 percent of emotional abuse of guidance school children (aged from 12 to 15) in Tehran. Vizeh et al. (20) reported that 49.46% of his study children had been exposed to emotional abuse, especially yelling and swearing, associating this extent of violence with the fact that their parents probably believed that disciplining children necessitated shouting and swearing.

It must be mentioned that the term “sexual abuse” did not necessarily and precisely, in this study, refer to sexual intercourse, but also included the use of language and touching to abuse the child. The girls, compared to the boys, were significantly more exposed to adult violence. Girls may be more susceptible to contempt, threats, and verbal abuse owing to their physical and psychological characteristics. This may partly account for their higher levels of vulnerability to sexual harassment.

### 5.1. Limitations

First, the questionnaire only assesses father’s violence against mother. Therefore, the actual rate of children’s exposure to domestic violence may be greater than that of the value obtained in this study. Second, this study was conducted only on the age group 15 to 16 years; therefore, generalization of the results to other age groups should be undertaken with caution.

### 5.2. Strong Points

Children’s exposure to different types of parental physical and psychological violence and their involvements in it, exposure to violence at school and in the community and media, existence of risk factors, and direct violence against children have also been measured. Findings of this study were based on self-rating of children, which could attain more realistic images compared to studies in which the parents assess the children’s exposure to domestic violence. The participants were children with normal conditions and lived at home with their families, not those who were referred to supportive centers afterwards. Thus, it can present a more realistic image of society.
